# Impact of a short-term pharmacy study abroad Program: student outcomes and program evaluation

**DOI:** 10.1186/s13104-024-06919-0

**Published:** 2024-10-02

**Authors:** Eman Mohamed Elmokadem, Azza A. Mahmoud, Harriet Bennett-Lenane, Kevin D. Murphy, Noha Khalil

**Affiliations:** 1 Department of Pharmacy Practice and Clinical Pharmacy, Faculty of Pharmacy, Future University in Egypt, Cairo, 11835 Egypt; 2 Department of Pharmaceutics and Pharmaceutical Technology, Faculty of Pharmacy, Future University in Egypt, Cairo, 11835 Egypt; 3 Pharmaceutical Care Group, School of Pharmacy, University College Cork, Cork, Ireland; 4 Department of Pharmacognosy and Medicinal Plants, Faculty of Pharmacy, Future University in Egypt, Cairo, 11835 Egypt

**Keywords:** Short-term study abroad programs, Program evaluation, Pharmacy students, International experience, Cultural experience

## Abstract

**Objective:**

This study examined the impact of a short-term study abroad program, focusing on program evaluation, attendee satisfaction, and acquired knowledge and skills. A questionnaire survey was conducted covering various aspects including demographics, program evaluation, and feedback.

**Results:**

Results indicated higher female participation due to gender imbalances in pharmacy students in Egypt, with senior students recognizing the value of international experience. Attendee satisfaction was high, with positive feedback on accommodation, tours, and workshop materials. Field visits and workshops provided valuable experiential learning, with attendees suggesting extending the program’s duration. The program equipped attendees with knowledge and skills relevant to pharmaceutical products and services, leading to improved competences and perceptions. The study concludes that such study abroad experiences profoundly impact personal growth and recommends integrating them into educational curricula for valuable experiences.

**Supplementary Information:**

The online version contains supplementary material available at 10.1186/s13104-024-06919-0.

## Introduction

Short-term study abroad programs (STSA) are increasingly recognized in education as valuable for extending learning beyond traditional classrooms and promoting cross-cultural interactions [1, 2]. Faculties of pharmacy are also offering international experiences to help students connect coursework to global healthcare systems and understand cultural nuances [3]. STSA courses for pharmacy students, lasting up to eight weeks [4], allow them to explore pharmacy practices worldwide under faculty guidance [5].

Participating in STSA programs offer various benefits [6]; including earning transferable credits, cultural immersion, language acquisition, cultural awareness, self-confidence, global identity strengthening, diversity understanding, flexibility, adaptability, independence, marketability enhancement, and preparation for international careers. Faculties of Pharmacy advocate for experiential learning opportunities such as study abroad, internships, research, and service learning to develop real-world skills related to global health issues and career goals.

Since 2016, the Faculty of Pharmacy at Future University in Egypt (FUE) has initiated an annual non-credit short-term study abroad program in Ireland for pharmacy students, in collaboration with University College Cork (UCC). Through lectures, field trips, and cultural experiences, students explore industrial pharmacy and pharmacy practice in Ireland. The program’s aim is to prepare pharmacists for the professional field by fostering student development, meeting individual professional interests, and teaching key skills relevant for pharmacists’ workplaces. To achieve this comprehensive aim, the program’s objectives include nurturing an appreciation for global cultures, facilitating comparisons between educational systems and pharmacy practices in Egypt and Ireland, and linking these experiences to future professional endeavors.

Existing research predominantly focuses on describing STSA programs and their perceived benefits, with limited empirical evidence on their specific impact on student learning outcomes. This lack of information is particularly concerning as pharmacy schools integrate these programs more frequently into their curriculum. To ensure STSA programs are truly beneficial and prepare students for the demands of a globalized healthcare field, a deeper understanding of their impact on learning outcomes is crucial. The primary outcome of this study is to inform management decision-making by providing evidence-based insights into the effectiveness of the STSA program. While enhancing student interest in study abroad is a potential benefit, the study’s primary focus is on evaluating the program’s impact on student learning and development.

Pharmacy education faces increasing pressure to prepare graduates for a globalized healthcare environment. While study abroad programs offer potential benefits, their effectiveness in achieving specific learning outcomes remains unclear. This study aims to evaluate the impact of a STSA program on pharmacy students’ knowledge, skills, and attitudes, and to identify areas for program improvement to optimize student outcomes and inform institutional decision-making.

## Methods

### Study design

To evaluate the impact of the STSA program on student learning outcomes and program satisfaction, a descriptive cross-sectional survey was conducted in October 2023. This timeframe followed the completion of the most recent two-week program, which has been offered annually as an elective summer course at the School of Pharmacy, UCC, Ireland, since 2016.

### Setting and participants

This study was conducted at the Faculty of Pharmacy at Future University in Egypt (FUE). Participants in the study abroad experience survey were students who met the following eligibility criteria: [1] Currently enrolled in the Faculty of Pharmacy at FUE, [2] Participated in the study abroad program to University College Cork (UCC), [3] 18 years of age or older, [4] In their third, fourth, or fifth year of the pharmacy program and [5] Provided informed consent. Students who did not meet these criteria or did not complete the survey were excluded from the study.

### Instrument development

A novel questionnaire was developed specifically for this study to assess participants’ perceptions of their study abroad experience. Given the exploratory nature of the research, descriptive statistics were primarily employed for data analysis.

### Instrument validation

To establish questionnaire reliability and validity, a rigorous development process was undertaken. Content validity was initially assessed through expert review involving ten faculty members with study abroad experience. Their feedback led to refinements in question clarity and content organization. Subsequently, a pilot test with fifteen students assessed the questionnaire’s face validity and enhanced the questionnaire’s comprehensiveness. Data collected from the pilot test were excluded from subsequent analyses.

These validation steps ensured the questionnaire accurately captured the intended constructs and provided reliable data for subsequent analysis.

### The final form of the survey

The final survey consisted of 19 main questions, with some including additional sub-questions, covering demographic data, general evaluation, personality development, sessions’ evaluations, instructor evaluations, advice for future trainees, and general comments. The survey used a mix of question formats, including multiple choice, rating scales, and open-ended questions, to gather a comprehensive range of data (A copy of the survey is available in a supplementary file).

### Ethical considerations

Participants signed informed consent for inclusion in this investigation. All research procedures adhered to the Helsinki Declaration for Human Subjects and Good Clinical Practice guidelines. This research obtained approval from the Research Ethics Committee of the Faculty of Pharmacy, FUE (REC-FPFUE-34/2023). Participants were provided with detailed information about the research’s objectives and procedures before participating. Only after confirming their understanding and providing explicit electronic consent were their responses recorded and included in the analysis and participants were informed about the confidentiality of their responses, and their anonymity was strictly maintained throughout the study.

#### Sample size justification

To achieve a 95% confidence level with a 5% margin of error in a 41 population of students who participated in the study abroad program to UCC, the required sample size was calculated to be approximately 93 students. This sample size ensures that the survey results accurately represent the population with the specified confidence and precision.

### Data collection

This research comprised FUE students who met the eligibility criteria, participated in a selection interview, and agreed to participate. After reviewing the cover letter, which explained the study’s purpose and their rights as participants, individuals who demonstrated understanding were invited to complete the questionnaire. The final version of the survey was distributed electronically by the primary investigator who was ready to address all questions. A total of 93 surveys were sent out, and 38 were completed and returned, yielding a satisfactory response rate of 40%.

### Data management and analysis

The collected data were reviewed, coded, tabulated, and entered into a computer using IBM SPSS Statistics for Windows, Version 29.0.2 (IBM Corp., 2023). Appropriate analyses were performed for each parameter based on the type of data obtained.

## Results

The analysis of participants’ responses sheds light on various aspects of the STSA program, offering insights into participant satisfaction levels, demographic trends, and the effectiveness of program elements. The satisfaction rates among participants ranged between 80% and 92%, indicating a generally positive perception of the program. The following sections delve into participant satisfaction across various program elements, ranging from pre-application orientation to field visits and skills acquisition. Additionally, participants’ feedback on program delivery methods, instructor effectiveness, session relevance, and overall program impact is presented and analyzed.

Analysis of participant demographics (Q1-3; Table 1) that focused on gender, age, and academic year of study revealed a higher participation rate among females in the program (68.42%). Furthermore, most program participants (42%) were enrolled in their final academic year (year five). Responses revealed that the most effective announcement method for the program (Q4) was through program announcements during lectures and labs (28.9%) followed by social media and faculty bulletin board (23.7%). Colleagues (15.8%) and other methods (7.9%) were reported as the least effective means of spreading the announcements.

**Table 1:** Participant demographics (questions 1–3)

	*n*	%*
**Gender**		
Male	12	31.57
Female	26	68.42
**Year of Study**		
Third	9	23.68
Fourth	13	34.21
Fifth	16	42.11
**Age (mean** ± SD)	21.26 ± 1.08 years	

The responses for the satisfaction of the participants for the STSA program showed overall satisfaction of 84.21% (Q5.m; Table 2). Attendees’ orientation with the training outline and program content was facilitated through a pre-application orientation and awareness session, ensuring they received concise and accurate information about the entire program (Q5.a; 81.58% satisfied).

**Table 2:** Satisfaction ratings for program elements

Question 5: How satisfied were you with the following?	Very satisfied		Satisfied		Neutral		Dissatisfied		Very Dissatisfied	
	n	%	n	%	n	%	n	%	n	%
a. Pre-application orientation and awareness about training outline and content	18	47.37	13	34.21	4	10.53	3	7.89	0	0.00
b. Accommodation	25	65.79	10	26.32	2	5.26	1	2.63	0	0.00
c. Welcome tour	24	63.16	10	26.32	3	7.89	1	2.63	0	0.00
d. Communication emails & messages	18	47.37	14	36.84	4	10.53	2	5.26	0	0.00
e. Transportation	16	42.11	15	39.47	2	5.26	3	7.89	2	5.26
f. Handouts and workshop materials	24	63.16	6	15.79	7	18.42	1	2.63	0	0.00
g. Field visits (AbbVie Inc., Boots Pharmacy, Cork University Hospital, Biopharma Training Centre)	24	63.16	9	23.68	3	7.89	2	5.26	0	0.00
h. Hand-on workshops	20	52.63	14	36.84	2	5.26	2	5.26	0	0.00
i. Final assessment and presentation for the students	21	55.26	13	34.21	3	7.89	0	0.00	1	2.63
j. Group mates	19	50.00	10	26.32	4	10.53	4	10.53	1	2.63
k. Program duration	15	39.47	11	28.95	6	15.79	2	5.26	4	10.53
l. Gained skills and experience	23	60.53	11	28.95	2	5.26	2	5.26	0	0.00
m. Overall satisfaction	23	60.53	9	23.68	4	10.53	2	5.26	0	0.00

Academic accommodations are vital in creating a supportive and comfortable learning environment for students. The attendees were satisfied (Q5.b; 92.11%) with the accommodation, which had excellent reviews from them regarding the good-sized clean rooms, a well-equipped kitchen, a spacious living area, amazing view and being close to the UCC campus.

The program attendees were also satisfied (Q5.c; 89.48%) with the welcome tour which included a tour of UCC campus, a visit to the Cork city hall and a city hall reception. Most of the attendees reported being satisfied (Q5.d; 81.58%) with the transportation offered by UCC. The availability of handouts and workshop material were reported in this survey as being satisfactory for the attendees (Q5.f; 78.95%).

During STSA program, field visits were carried out to different training sites and showed 86.84% satisfaction percentage (Q5.g). About 90% of the students were satisfied with the held workshops (Q5.h). The final quiz and presentation were effective assessment methods in the training program (Q5.i; 89.47% satisfied). Most of the attendees were satisfied with their group mates and only 4 students (Q5.j; 10.53%) reported that they were dissatisfied.

Concerning the program’s duration (two-weeks), the attendees reported that they were satisfied with the program duration (Q5.k; 68.42%) and that it was sufficient to fulfill the program’s objectives, however minority of the attendees (15.79%) reported that they wanted to increase the duration of the program for more site visits (Q6); “It needed to have more time,” noted one student “but overall, it was great”. Most of the attendees (Q5.l; 89.48%) reported that regarding the gained skills and experience, they had gained a lot of skills either on the personal, academic, or social level.

The findings of attendees’ perceptions regarding the learning outcomes (Q7; Table 3) showed that the program managed to equip the attendees with the knowledge and skills (Q7.b & g; almost 90% satisfaction) to deliver pharmaceutical products and quality pharmacy services. STSA impacted the ability of the attendees in a positive way to work in a team through team-based learning (TBL) which allowed them to coordinate efforts with colleagues in a constructive way (Q7.c). Additionally, it enabled them to explore new academic interests and become more confident in their abilities and they also learned how valuable is time management and practiced punctuality (Q7.a & d). Attendees had the opportunity to practice language skills in everyday interactions, reinforcing their studies (Q7.f). Also, STSA program allowed them to explore new cultures and that it was a rewarding and transformative experience that offers numerous personal and professional benefits that helped them broaden their horizons and become more career oriented (Q7.e & h).

STSA attendees gained essential skills including promptness, time-management, and insights into European healthcare guidelines (Q8). The survey results of attendees’ perceptions regarding program delivery indicate that about 80% of the respondents strongly agree or agree with the effectiveness of the program delivery method and content (Q9; Table 3). Around 80% of attendees found that workshops and sessions in the STSA program, covering areas like the pharmaceutical industry and clinical pharmacy (Q10; Table 4), were relevant to the program aim extending their learning into real-life experiences and preparing pharmacists for the professional field.

**Table 3:** Perceptions regarding learning outcomes, program delivery, and instructors

Question 7: What is your perception regarding the following?		Strongly agree					Agree							Neutral							Disagree						Strongly disagree						
		n			%		n				%			n				%			n			%			n			%			
a. The program has helped me to become more confident in my abilities		22			57.89		11				28.95			3				7.89			2			5.26			0			0.00			
b. I gained new knowledge from the program		21			55.26		14				36.84			2				5.26			1			2.63			0			0.00			
c. The program has impacted my ability to work in a team		20			52.63		12				31.58			4				10.53			2			5.26			0			0.00			
d. I learned time management & punctuality		19			50.00		13				34.21			5				13.16			1			2.63			0			0.00			
e. I acquired intercultural communication skills		27			71.05		6				15.79			3				7.89			2			5.26			0			0.00			
f. My foreign language has improved		21			55.26		8				21.05			6				15.79			1			2.63			2			5.26			
g. I developed new personal skills such as independence and problem-solving		22			57.89		12				31.58			3				7.89			1			2.63			0			0.00			
h. Studying abroad experience has broadened my horizons, made me ambitious and more career-oriented		23			60.53		13				34.21			2				5.26			0			0.00			0			0.00			
**Question 9**: What is your perception regarding the program delivery method and content?		Strongly agree					Agree							Neutral							Disagree						Strongly disagree						
		n			%		n				%			n				%			n			%			n			%			
a. Ability to link the training material to the practical part		14			36.84		18				47.37			3				7.89			2			5.26			1			2.63			
b. Use of audio-visual training aids & techniques		14			36.84		15				39.47			5				13.16			3			7.89			1			2.63			
c. Hands-on activities were satisfactory		15			39.47		14				36.84			7				18.42			1			2.63			1			2.63			
d. Availability of learning resources & handouts		16			42.11		16				42.11			5				13.16			0			0.00			1			2.63			
e. The pace & tone were acceptable to facilitate learning		17			44.74		12				31.58			8				21.05			1			2.63			0			0.00			
**Question 12**: What is your perception regarding the training instructors		Strongly agree					Agree							Neutral							Disagree						Strongly disagree						
		n			%		n				%			n				%			n			%			n			%			
a. Instructors encouraged interaction & team-working		20			52.63		13				34.21			4				10.53			1			2.63			0			0.00			
b. Instructors were always available to answer questions		20			52.63		14				36.84			3				7.89			1			2.63			0			0.00			
c. Instructors were knowledgeable & experienced		24			63.16		10				26.32			3				7.89			1			2.63			0			0.00			
d. Instructors supported me to learn & get more knowledge		20			52.63		13				34.21			4				10.53			1			2.63			0			0.00			

**Table 4:** Perceived relevance of program sessions to preparing students for the professional field

Question 10: Which sessions did you find most relevant to the program aim (prepare pharmacists for the professional field)?	Not relevant		Relevant		Very relevant		Did not attend	
	n	%	n	%	n	%	n	%
a. Library workshop	5	13.16	19	50.00	9	23.68	5	13.16
b. Boots Pharmacy-Cork Half Moon Street Visit	2	5.26	13	34.21	17	44.74	6	15.79
c. Communication Skills/Cultural Diversity Workshop	4	10.53	13	34.21	15	39.47	6	15.79
d. AbbVie Inc. Visit	0	0.00	11	28.95	20	52.63	7	18.42
e. Biopharma Training Centre Visit	1	2.63	16	42.11	13	34.21	8	21.05
f. Coating Extrusion Workshop	0	0.00	16	42.11	16	42.11	6	15.79
g. Tableting and Compression – Practical	0	0.00	13	34.21	16	42.11	9	23.68
h. Good Manufacturing - Hand-on Experience	1	2.63	15	39.47	13	34.21	9	23.68
i. Aseptic Processing Workshop	0	0.00	12	31.58	18	47.37	8	21.05
j. Freeze-Drying Session	0	0.00	16	42.11	13	34.21	9	23.68
k. Respiratory Session	1	2.63	16	42.11	14	36.84	7	18.42
l. Adrenaline Auto-Injector Workshop	1	2.63	14	36.84	17	44.74	6	15.79
m. Dysphagia Workshop	1	2.63	16	42.11	23	60.53	9	23.68
n. Reviewing Respiratory Cases in Cork University Hospital	2	5.26	15	39.47	14	36.84	7	18.42
o. Hepatic Diseases Tutorial with Hospital Pharmacist	2	5.26	14	36.84	15	39.47	7	18.42
p. Respiratory Disease Workshop / Health Promotion – Smoking Cessation	1	2.63	15	39.47	14	36.84	8	21.05
q. Cardiovascular Disease Workshop	1	2.63	14	36.84	18	47.37	5	13.16

Attendees’ comments suggested that specific sessions, like the hospital visit, required more time (Q11). Additionally, they expressed the need for increased interaction with other healthcare team members. “The hospital visit was very important to me as I managed to interact with clinical pharmacists and critical care team as well, and I would have loved to spend more time with them” mentioned an attendee.

In this STSA, approximately 90% of the attendees strongly agreed or agreed that the training instructors were effective (Q12; Table 3) as they were readily available for assistance, maintained professionalism, and actively encouraged interaction and engagement. In the program, instructors utilized engaging tools to encourage active learning and teamwork, fostering the development of essential team-working skills among program attendees (Q12.a). The instructors at STSA possessed extensive knowledge and experience, providing valuable support to the attendees in their learning journey and facilitating the acquisition of additional knowledge (Q12.b, c & d). The program attendees commented that instructors were always available for help, professional and encouraged interaction and engagement (Q13).

Attendees recommended that future trainees must be responsible enough to comply with the duties assigned by the instructors as well as accepting feedback from the instructors and colleagues, which will help improve their attitude and always keep a good relationship with colleagues (Q14). Moreover, others stressed the importance of punctuality and being on time as this affects the whole group. The attendees advised their colleagues to “Manage your budget wisely” and taking care that “No need to pack a lot of clothing.”

The program attendees found the level of work and difficulty of the sessions’ content to be comparable to what they were accustomed to at their home university (52.6%). Conversely, 26.3% found it more challenging, while 21.1% found it less challenging.

Trips in the program helped the attendees to immerse themselves in a foreign culture which furthers awareness of their own cultural perspectives and helped them develop cross-cultural communication skills which is a highly valuable ability in a globalized world.

The overall academic experience helped the attendees acquire skill-based training which facilitated expanding their horizons and opened a world of new educational opportunities (Q17). The attendees reported positive feedback as this program helped them become more self-sufficient, forced them out of their comfort zone and challenged them to adapt to new situations (Q18). Moreover, they reported that it helped them experience a different way of life, which broadened their horizons and helped them appreciate diversity, which provided them with a better understanding of the global world.

As a summary for the results, students were overwhelmingly positive about the program, with a strong majority (84%) reporting high satisfaction. This satisfaction extended to various aspects of the experience, including the pre-application orientation (over 81% satisfied), comfortable accommodation (over 92% satisfied), and well-received workshops (nearly 90% satisfied). While nearly all participants found the instructors effective, some suggested incorporating more interaction with healthcare professionals during hospital visits (around 16% requested improvement). Overall, the program appears to have successfully equipped attendees with valuable knowledge and skills, promoting personal growth alongside academic learning, with a small percentage (around 16%) desiring a longer program with more site visits. All the STSA attendees reported that they will recommend the program to other colleagues stating that it is one of the best investments they can make for their future (Q19).

## Discussion

STSAs are a quickly expanding subset of education nowadays. They are intended to give pharmacy students an overview of pharmacy practice in different countries. Through these encounters, students are able to understand the significance of culture in other healthcare systems and relate their curriculum to a larger global framework [2].

The STSA evaluation survey responses denoted higher participation rate among females in the program which may be possibly due to existing gender imbalances among pharmacy students in Egypt. Previous research has noted 2 to 3 times higher proportion of female pharmacy students compared to male students in Egypt [7]. Additionally, participation in this program was limited to students who were enrolled in level 3 or higher within the faculty. As third year students completed a substantial portion of their courses; therefore, they possess the necessary academic foundation to derive maximum benefit from an overseas program. Also, senior faculty students showed the highest participation percentage in this program as they recognize the significance of international experience in the global job market, understanding that it provides a competitive edge by showcasing adaptability, cross-cultural communication skills, and a broader worldview to potential employers [8]. Furthermore, within the Egyptian culture, parents often prefer their children to travel abroad when they develop a sense of independence, making them better equipped to handle the challenges and responsibilities that come with studying abroad.

Effective announcement methods were utilized to ensure broad awareness of the program [9]. In this study, announcements during lectures and labs were found to be the most effective method due to the captive audience and focused environment during lectures and labs, which increases the likelihood of students noticing and remembering the announcement. The initial recruitment in the program process was based on meeting specific requirements, however there were some challenges that faced some students who failed to adhere to schedules, highlighting the importance of punctuality in fostering professionalism and efficiency within the learning environment.

Reinforcing experiential and relational learning is the key goal of conducting field visits [10]. They are a way of improving learning experience by creating links to the real world [11]. During STSA program, field visits were carried out to different training sites where they reinforce experiential and relational learning and help the attendees acquire hands-on learning opportunities, practice different roles of the pharmacist as well as providing them with practical exposure to different pharmacy careers which will help them in their future choice of pharmacy career pathways.

The program managed to equip the attendees with the knowledge and skills to deliver pharmaceutical products and quality pharmacy services. Recently, there has been an increase in awareness of the importance of experiential training [12] and this was fulfilled through STSA program. In another note, the experiential training component of pharmacy programs needs to be redesigned in order to support the profession’s shift from drug dispensers to medication therapy managers [13]. Therefore, to enhance the moving forward in this program in the coming years, it is crucial to expand both the quantity and quality of community and hospital pharmacy practice sites available for experiential training.

It is highly recommended to engage trainees through interactive activities and encourage interaction among them [14]. In this regard, the program sessions were delivered through various methods, which included providing students with hard copies of training materials, utilizing promotional videos and practical tools during training sessions, incorporating hands-on activities, and conducting site visits. The attendees agreed with the effectiveness of the program delivery method and content which suggests that the chosen approaches were effective and well-received by most of the attendees. Furthermore, STSA impacted the ability of the attendees in a positive way to work in a team through TBL. This comprehensive teaching approach for healthcare education is characterized by an interactive classroom setting where students collaborate in groups to discuss and apply what they have learned to real-world clinical situations [15–17]. Moreover, several workshops were held during the STSA program including dysphagia, liver diseases, respiratory, cultural awareness, adrenaline administration, extrusion and spheronization coating as well as tableting and compression workshop. These workshops successfully introduced new concepts to the attendees, spurring them to investigate it further on their own and encouraged the practice of actual methods where it was a great way to teach hands-on skills. The workshops in the program were most relevant to the program aim to facilitate the attendees shift towards patient-centered care and to equip attendees to address complex drug therapy needs and prepare them effectively for professional healthcare roles and job market competitiveness post-graduation. In addition, being surrounded by a foreign language environment is a highly effective method for learning a new language [18], as supported by feedback from participants.

The evaluation of instructors in the field of education is widely recognized to enhance their effectiveness [19–21]. Patient safety experts emphasize the importance of communication and teamwork skills in delivering quality healthcare. Effective collaboration among clinical and nonclinical staff can lead to improved patient outcomes, error prevention, increased efficiency, and enhanced patient satisfaction [22]. Attendees’ questions during the programs’ sessions played a crucial role in meaningful learning and scientific inquiry [23]. Moreover, expert instructors demonstrate a high level of responsiveness to their students, prioritizing their needs over bureaucratic controls such as curriculum, assessment, and time constraints [24]. The program instructors were always available. This feedback holds great significance, as the quality of instructors stands as the primary factor related to educational programs that significantly influences attendee achievement [22].

The Faculty of Pharmacy at FUE is renowned for its exceptional quality of education (accreditation by the Egyptian accreditation system NAQAAE and the American accreditation system ACPE). By obtaining accreditation from both national and international bodies, the Faculty of Pharmacy at FUE demonstrates its dedication to providing students with a high-quality learning experience that aligns with global educational standards. Consequently, the program participants noted that the workload and the complexity of the session content were on par with what they typically encountered at their home university.

The trips in the program helped the attendees to immerse themselves in a foreign culture which give endless opportunities to improve personal development, such as confidence, self-esteem, leadership, and independent thinking [25, 26]. A variety of non-academic trips were included in the STSA program. Such trips that engage students through enjoyable activities can significantly enhance the educational value of the program. These experiences have the potential to create lasting and meaningful episodic memories that are closely tied to the program and the visited country, impacting not only their learning but also their lives [27]. Also, A recent study by Goldstein (2022) focused specifically on intercultural outcomes, analyzing data from 68 studies [28].

The final quiz and presentation in the program allowed attendees to measure their knowledge gain and align it with the training objectives. Building on this foundation, STSA attendees gained essential pharmacy practice skills. These skills included essential pharmacy practice skills that would help them to compare the national guidelines they learnt in their country by guidelines in other countries and thus, apply optimal practice if they work in the clinical field. They also developed teamwork, adaptability, resilience, and research skills, enhancing their professional capabilities, and preparing them for diverse challenges in their careers. Some of them commented that they felt that life abroad was not as hard as they thought. In addition, while attendee satisfaction with their group members suggests a positive program experience, it also highlights a potential challenge – navigating diverse personalities. This challenge, however, presented an opportunity for attendees to develop skills in interpersonal communication and conflict resolution.

The attendees reported that they wanted to increase the duration of the program, and this showed how they were enjoying the program and wanted to increase the benefit gained. Extended study abroad influences language use, academic success, personal development, and career choices, emphasizing the importance of short-term programs lasting at least 6 weeks [29].

From the findings of this study, it is recommended for future program to utilize its announcements during lectures and labs as it was reported to be the most effective method for reaching a wider student audience, moreover, to implement applicant interviews before applying to the program to assess responsibility skills and meet the evaluated program requirements. It is also important for future programs to prioritize the expansion of both the quantity and quality of community and hospital pharmacy practice sites for enhanced experiential training opportunities and finally to extend the program’s duration from two weeks to at least six weeks to allow for visits to more training sites.

### Limitations

The study had several limitations. Firstly, it focused on a single collaboration between two institutions. Secondly, there was a small response rate from participants in the survey, although this represented 40% of the program’s participants. The low number of participants in the program was attributed to the cost burden some students faced. Thirdly, recall bias may occur in this survey for students who attended the program for a long period, as the participants attended the program between 2016 and 2023. Despite these limitations, our research offers fresh perspectives on how pharmacy students view their study abroad experience and includes recommendations to enhance the program in the future and increase participant numbers.

## Conclusion

The primary objective of the STSA program was to equip pharmacy students with the knowledge and skills necessary for successful careers. By exposing students to diverse healthcare systems and practical experiences, the program aimed to inform administrative decision-making. The evaluation revealed that the program effectively met these goals, as evidenced by positive student feedback and the development of essential competencies.

While the study demonstrated the program’s efficacy, limitations such as sample size and potential recall bias necessitate further investigation. Future research should explore the program’s long-term impact, investigate factors influencing student participation, and replicate the study in different settings. By addressing these areas, the STSA program can be continuously improved to better prepare students for the complexities of the global healthcare landscape.

Ultimately, the STSA program has proven to be a valuable experience for participants, fostering personal and professional growth. As the world becomes increasingly interconnected, programs like these are essential for cultivating globally competent healthcare professionals.

## Electronic supplementary material

Below is the link to the electronic supplementary material.

Supplementary Material 1

## Data Availability

The data that support the findings of this study are available from the corresponding author, upon reasonable request.

## Declarations

### Generative AI and AI-assisted technologies in the writing process’

During the preparation of this work the author(s) used ChatGPT and Gemini to improve the manuscript readability and language. After using this tool/service, the author(s) reviewed and edited the content as needed and take(s) full responsibility for the content of the publication.
