# Supplementary material for: Impact of a short-term pharmacy study abroad Program: student outcomes and program evaluation
Source: BMC Res Notes. 2024 Oct 2;17:285. doi: 10.1186/s13104-024-06919-0 (PMC11446069; doi:10.1186/s13104-024-06919-0)
Supplement: Supplementary file 1 — Supplementary Material 1 [file 13104_2024_6919_MOESM1_ESM.docx]

**Short Term Study Abroad Program Evaluation Survey**

Thank you for participating in UCC short-term study abroad program. We hope you had a fruitful and beneficial experience.

We invite you to participate in a research survey that has been established by Future University in Egypt.

The purpose of this research is to understand the impact and extent of benefit of short-term study abroad (STSA) program, and to define the key points that would guarantee its success.

The survey will take approximately 20 minutes.

Involvement in this study is completely voluntary, and the information collected will remain confidential.

Your responses will only be reported in aggregate form and will only be used for research purposes.

Your anonymity will also be safeguarded in any publication of the results of this research, and no company or individual names will be attributed to any results that are discussed or written up.

You might not directly benefit from participation in this study, but the results could be beneficial to others.

This project has been approved by the Faculty of Pharmacy at the Future University in Egypt Ethics Committee.

By clicking "next" you agree to participate in this study.

**Survey questions**

| No. | Question |
| --- | --- |
| Q1 | Gender |
| Q2 | Age |
| Q3 | Year of study |
| Q4 | How did you know about the program |
| Q5 | How satisfied were you with the following |
|  | 1. Pre-application orientation and awareness about Training outline and content |
|  | 1. Accommodation |
|  | 1. Welcome Tour |
|  | 1. Communication emails & messages |
|  | 1. Transportation |
|  | 1. Handouts and workshop material |
|  | 1. Field visits (Abbvie, Boots, Hospital, Training center) |
|  | 1. Workshops |
|  | 1. Final Assessment and Presentation |
|  | 1. Group mates |
|  | 1. Program Duration |
|  | 1. Gained Skills and Experience |
|  | 1. Overall Satisfaction |
| Q6 | Additional feedback on logistics |
| Q7 | What is your perception regarding the following? |
|  | 1. The program has helped me to become more confident in my abilities |
|  | 1. I gained new knowledge from the program |
|  | 1. The program has impacted my ability to work in a team |
|  | 1. I learned time management & punctuality |
|  | 1. I acquired intercultural communication skills |
|  | 1. My foreign language has improved |
|  | 1. I developed new personal skills such as independence and problem-solving |
|  | 1. Studying abroad experience has broadened my horizons, made me ambitious and more career-oriented |
| Q8 | Any other skills you feel you gained during the program? |
| Q9 | What is your perception regarding the program content? |
|  | 1. Ability to link the training material to the practical part |
|  | 1. Use of audio-visual training aids & techniques |
|  | 1. Hands-on activities were satisfactory |
|  | 1. Availability of learning resources & handouts |
|  | 1. The pace & tone were acceptable to facilitate learning |
|  | 1. Scheduled activities were enriching & relevant |
| Q10 | Which sessions did you find most relevant to the program aim (prepare pharmacists for the professional field)? |
|  | 1. Library workshop |
|  | 1. Visit to Boots Pharmacy Half Moon Street |
|  | 1. Workshop on Communication Skills/Cultural Diversity |
|  | 1. Site Visit Abbvie |
|  | 1. Tour of Biopharma Training Centre |
|  | 1. Workshop on Coating Extrusion |
|  | 1. Tableting and Compression – Practical |
|  | 1. Workshop on Good Manufacturing Practice |
|  | 1. Aseptic Processing |
|  | 1. Freeze-Drying |
|  | 1. Respiratory Workshop |
|  | 1. Adrenaline Auto-Injector Workshop |
|  | 1. Dysphagia Workshop |
|  | 1. Workshop on Reviewing Respiratory Cases in Cork University Hospital |
|  | 1. Hepatic Diseases Tutorial with Hospital Pharmacist |
|  | 1. Respiratory Disease Workshop / Health Promotion – Smoking Cessation |
|  | 1. Cardiovascular Disease Workshop |
| Q11 | Any additional comments regarding the sessions? |
| Q12 | What is your perception regarding the training instructors? |
|  | 1. Instructors encouraged interaction & team-working |
|  | 1. Instructors were always available to answer questions |
|  | 1. Instructors were knowledgeable & experienced |
|  | 1. Instructors supported me to learn & get more knowledge |
| Q13 | Any additional comments regarding the instructors? |
| Q14 | Advice for future trainees |
|  | 1. Average pocket money needed |
|  | 1. What to pack |
|  | 1. What not to pack |
|  | 1. Places to eat or buy groceries cheaply |
|  | 1. Places to go on the weekend |
| Q15 | How did the level of work and difficulty of sessions' content compare to what you are used to at your home University? |
| Q16 | What’s your evaluation for the trips? |
| Q17 | Do you have any comments on your overall academic experience? Consider differences in teaching style, expectations, and direct enrollment. |
| Q18 | Do you have any comments on your study abroad experience? Please include your responsibilities as well as any positive or negative feedback. |
| Q19 | Would you recommend this program to your colleagues? |
